# Proliferative capacity exhibited by human liver-resident CD49a+CD25+ NK cells

**DOI:** 10.1371/journal.pone.0182532

**Published:** 2017-08-09

**Authors:** Glòria Martrus, Tobias Kautz, Sebastian Lunemann, Laura Richert, Laura Glau, Wilhelm Salzberger, Hanna Goebels, Annika Langeneckert, Leonard Hess, Tobias Poch, Christoph Schramm, Karl J. Oldhafer, Martina Koch, Eva Tolosa, Björn Nashan, Marcus Altfeld

**Affiliations:** 1 Heinrich Pette Institute, Leibniz Institute for Experimental Virology, Hamburg, Germany; 2 Univ. Bordeaux, Inserm, Bordeaux Population Health Research Center, team SISTM, UMR1219 and Inria, Bordeaux, France; 3 Department of Immunology, University Medical Center Hamburg-Eppendorf, Hamburg, Germany; 4 First Department of Medicine, University Medical Center Hamburg-Eppendorf, Hamburg, Germany; 5 Department of General & Abdominal Surgery, Asklepios Hospital Barmbek, Semmelweis University of Medicine, Asklepios Campus, Hamburg, Germany; 6 Department of Hepatobiliary and Transplant Surgery, University Medical Center Hamburg-Eppendorf, Hamburg, Germany; University of Sydney, AUSTRALIA

## Abstract

The recruitment and retention of Natural Killer (NK) cells in the liver are thought to play an important role during hepatotropic infections and liver cirrhosis. The aims of this study were to determine differences between liver-derived and peripheral blood-derived NK cells in the context of liver inflammation and cirrhosis. We conducted a prospective dual-center cross-sectional study in patients undergoing liver transplantation or tumor-free liver resections, in which both liver tissue and peripheral blood samples were obtained from each consenting study participants. Intrahepatic lymphocytes and PBMCs were stained, fixed and analyzed by flow cytometry. Our results showed that, within cirrhotic liver samples, intrahepatic NK cells were particularly enriched for CD49a+ NK cells when compared to tumor-free liver resection samples. CD49a+ liver-derived NK cells included populations of cells expressing CD25, CD34 and CXCR3. Moreover, CD49a+CD25+ liver-derived NK cells exhibited high proliferative capacity *in vitro* in response to low doses of IL-2. Our study identified a specific subset of CD49a+CD25+ NK cells in cirrhotic livers bearing functional features of proliferation.

## Introduction

Lymphocytes in the liver consist of liver-resident cells as well as lymphocytes circulating through the liver from the portal vein and the hepatic artery [1, 2]. Liver-resident type 1 innate lymphoid cells (ILC1s), including Natural Killer (NK) cells, have been suggested to regulate liver fibrosis during chronic hepatotropic infections and chronic inflammatory processes. NK cells are classified according to their CD56 marker expression levels in CD56^bright^, CD56^dim^ and CD56^-^CD16^+^ NK cells and represent an enriched population within the human intrahepatic lymphocytes (IHLs) by constituting up to 40% of this population [3–5]. Data obtained in mouse models using parabiotic experiments have defined liver-resident NK cells as being CD49a+DX5- [6]. Furthermore, it was demonstrated that these liver-resident NK cells represent a distinct lineage from bone marrow-derived NK cells, and might have originated from hepatic progenitor cells during fetal development [7]. A number of recent studies have characterized liver-resident and tissue-resident NK cells in mouse models and humans using several surface markers, including CD49a [6, 8], DNAM-1 [9], CXCR6 [10–12], CCR5 [12], CD103 [7, 13] CD49e [14] or transcription factors including T-bet [10, 15], Eomes [10, 15] or PLZF [16]. These studies have defined liver-resident NK cells depending on specific transcription factors or integrin receptors. While our understanding of the phenotypical properties of liver-resident NK cells has been advanced by those studies, there is still some lack of knowledge about the functional capacities of liver-resident NK cells.

In our study, we have focused on several activation (CD25) and differentiation (CD34) markers, as well as integrin receptors (CD49a and CXCR3) to study the maturation and homing capacities of liver-resident NK cells. Our aim was to determine whether those markers would be especially expressed on liver-resident CD49a+ NK cells. Altogether, we demonstrate that liver-resident CD49a+ NK cells in humans with advanced liver disease include subsets of CD25+ proliferating cells responding to low doses of exogenous IL-2 as well as NK cell subsets expressing CXCR3, the receptor for IP-10, and CD34, a marker for progenitor NK cells. The results suggest that subsets of human liver-resident NK cells retain distinct functional characteristics including proliferation capacities and persistence and might contribute to liver inflammation and fibrosis.

## Materials and methods

### 1. Study design and study population

We conducted a prospective cross-sectional study in adult patients undergoing liver transplantation at the University Medical Center Hamburg-Eppendorf (UKE). Both explant liver tissue and peripheral blood samples were obtained from each consenting study participant undergoing a transplantation procedure. All consenting adult participants (age over 18 years) undergoing liver transplantation during the study period were eligible. We furthermore obtained tumor-free liver tissue and peripheral blood samples from a cohort of individuals undergoing surgical liver resection due to hepatocellular carcinoma at the Asklepios Hospital Barmbek. Healthy liver tissue from the tumor surrounding areas was excised. All study participants provided written informed consent, according to the ethical guidelines by the Institutional Review Board of the medical faculty at the University of Hamburg that approved the study protocol. The demographics and clinically characteristics of study subjects are summarized in Tables 1 and 2.

**Table 1:** Demographics and clinical characteristics from explanted liver tissue samples.

	**Total number of individuals**	**19**			
	Sex (f/m; %f/%m)	7/12; 37%, 63%			
	age (years; range)	57; 38–69			
**Primary liver disease**	HCV (n°) *	6			
	PSC (n°)	2			
	ALD (n°)	6			
	HCC (n°)	1			
	cholangiocarcinoma (n°)	1			
	overlap syndrome AIH/PBC or AIH/PSC (n°)	2			
	polycystic liver disease (n°)	1			
**Clinical Data**	CMV (neg/pos; %neg/%pos)	13/6; 68%/32%			
		**Males**	**Females**	**RV Males**	**RV Females**
	MELD (median /min-max)	12.15 (7–35)	19.7 (7–29)		
	INR (median /min-max)	1.26 (0.96–2.2)	1.34 (1–1.89)		
	Creatinine (mg/dl) (median /min-max)	1.18 (0.58–3.8)	1.2 (0.57–4.8)	0.6–1.3	0.5–1
	Thrombocytes (/μl (x 1000)) (median /min-max)	109 (25–201)	62 (29–210)	150–400	150–400
	Bilirubin (mg/dl) (median /min-max)	2.2 (0.3–22.9)	4.5 (0.2–22)	<1.2	<1.2

HCV: Hepatitis C Virus infection, PSC: Primary Sclerosing Cholangitis, ALD: Alcoholic Liver Disease, HCC: Hepatocellular carcinoma, AIH: Autoimmune Hepatitis, PBC: Primary Biliary Cholangitis, INR: International Normalized Ratio, MELD: Model For End-Stage Liver Disease, CMV: Cytomegalovirus status.

*5 out of HCV+ livers have developed HCC as a complication of the cirrhosis. RV: Reference Values.

**Table 2:** Demographics and clinical characteristics from liver resection samples.

	**Total number of individuals**	**5**		
	Sex (f/m; %f/%m)	3/2; 60%, 40%		
	age (years; range)	65; 56–73		
**Clinical Data**	CMV (neg/pos)	1/3, 1ND		
		**Males**	**Females**	**Reference values**
	INR (median /min-max)	1.08 (1–1.16)	1.05 (0.95–1.22)	
	Creatinine (mg/dl) (median /min-max)	0.8 (0.7–0.9)	0.7 (0.6–1.2)	0.6–1.1
	Thrombocytes (/μl (x 1000)) (median /min-max)	205.5 (130–281)	340 (130–344)	150–370
	Bilirubin (mg/dl) (median /min-max)	0.55 (0.3–0.8)	0.7 (0.6–0.8)	<1.2

INR: International Normalized Ratio, CMV: Cytomegalovirus status. ND: non-determined

### 2. Cell preparation

Intrahepatic lymphocytes (IHLs) were isolated following a hashing protocol established in our laboratory. Briefly, 10 to 20 grams (g) of liver were sliced into small pieces. Tubes containing 3 g of sliced tissue and 3 ml of RPMI+10% FBS (R10) were hashed at room temperature using the gentleMACS^™^ Octo Dissociator (Miltenyi Biotec, Germany). The recovered tissue was successively strained through 100μm, 70μm and 40μm Easystrainer^™^ filters (Greiner Bio-One GmbH). Blood samples from the same study participant obtained pre-surgery were processed by Ficoll-gradient purification to gain peripheral blood mononuclear cells (PBMCs). The recovered cells were immediately processed for flow cytometry analysis (FACS).

### 3. Antibody staining and flow cytometry

Monoclonal antibodies anti-CD56 (BUV395, clone NCAM16.2, BD Horizon^™^), anti-CD3 (BUV737, clone UCHT1, BD Horizon^™^), anti-CD25 (PE-Cy7, clone M-A251, Biolegend), anti-CD34 (PE-CF594, clone 581, Biolegend), anti-CD49a (PE, clone TS2/7, Biolegend), anti-CD16 (BV786, clone 3G8, Biolegend), anti-CD45 (BV711, clone HI30, Biolegend), anti-CXCR4 (BV605, 12G5, Biolegend), anti-CD14 (BV510, clone M5E2, Biolegend), anti-CD19 (BV510, clone HIB19, Biolegend), anti-CXCR3 (APC-Cy7, clone G025H7, Biolegend), anti-NKG2C (AF700, clone 134591, R&D Systems), anti-DNAM-1 (APC, clone 11A8, Biolegend), anti-CD57 (FITC, clone HNK-1, Biolegend) and Zombie Aqua staining (Biolegend) were used. Freshly isolated cells were washed, stained at a final volume of 100μl with PBS and incubated for 20 minutes at room temperature. Cells were subsequently washed and fixed with 4% paraformaldehyde for 20 minutes. Samples were acquired on a LSR Fortessa (BD Biosciences) and results were analyzed using FlowJo software version 10.

### 4. Cell sorting and proliferation assay

Freshly isolated IHLs were stained using: CD3 (PE/Dazzle 594, clone UCHT1, Biolegend), CD25 (PE-Cy7, clone M-A251, Biolegend), CD16 (BV786, clone 3G8, Biolegend), CD45 (BV711, clone HI30, Biolegend), CD56 (BV605, clone NCAM, Biolegend), CD14 (BV510, clone M5E2, Biolegend), CD19 (BV510, clone HIB19, Biolegend), CD49a (FITC, clone TS2/7, Biolegend) and Zombie Aqua (Biolegend) and, after lineage gating, 4 populations (CD49a+CD25+, CD49a+CD25-, CD49a-CD25+ and CD49a-CD25-) were sorted using a BD FACSAria^™^ Fusion. Subsequently proliferative capacity of the sorted cells was assessed using a CFSE proliferation assay. Briefly, cells were resuspended in PBS+2% FBS and CellTrace^™^ CFSE (Thermofisher Scientific) was added at a 1μM final concentration. Cells were left in the dark for 10 minutes and 1ml of FBS was added to stop the staining. After 1 minute incubation, 5ml R10 were added on top and cells were incubated for 5 extra minutes. A washing step was performed and cells were resuspended at 30,000 cells/ml in R10 + 30UI/ml of IL-2 and left in the incubator at 37°C. CFSE fluorescence was measured using a LSR Fortessa (BD Biosciences) on day 7.

### 5. t-SNE analysis

The t-Distributed Stochastic Neighbor Embedding (t-SNE) [17] analysis was performed in R using the packages flowCore [18] and Rtsne [19]. For the t-SNE analysis, gated events representing living NK cells (CD56+ CD16+/-) from liver and peripheral blood from 19 patients were independently merged and randomly subsampled to 100,000 events for comparative purposes. Subsequently, liver NK cell and peripheral NK cell files were combined into one single FCS file containing 200,000 events. Fluorescence channels were then scaled according to the logicle display method [20] and t-SNE analysis was run considering the markers DNAM1, CXCR3, NKG2C, CD56, CXCR4, CD16, CD57, CD49a, CD25, CD34 and CXCR6. After analysis, the results were plotted using the R package ggplot2 [21] into merged plots of liver and PBMCs and into plots containing cells derived from only liver or only PBMCs, respectively. Additionally, liver cells were split into CD49a+ and CD49a- cells for plotting t-SNE maps.

### 6. Statistical methods

Percentages of cell sub-populations within the same individual were compared between PBMC and IHLs, and between CD49a+ vs CD49a- cell populations within IHLs using Wilcoxon signed rank tests. Test multiplicity was controlled by a false discovery rate (FDR) procedure accounting for dependency among statistical tests [22]. FDR-adjusted p-values <0.05 were considered statistically significant. Statistical analyses were done with SAS, version 9.3 (SAS Institute, Cary, North Carolina, USA).

## Results

### Unsupervised analysis revealed profound phenotypic differences between peripheral blood and intrahepatic NK cells

Human liver-resident NK cells have previously been shown to carry a distinct phenotype compared to peripheral blood NK cells using a limited number of parameters [23]. In particular, CD49a expression has been described to define liver-resident NK cells in mouse models [6, 23] as well as in human liver samples [8, 24]. We analyzed a set of surface markers, including cytokine receptors, integrin receptors and activation markers in matched liver and blood samples from 19 individuals undergoing liver transplantation at the Department of Hepatobiliary and Transplant Surgery of the UKE and from 5 individuals undergoing surgical liver resection at the Department of General & Abdominal Surgery of the Asklepios Hospital Barmbek. The demographics and clinical characteristics of study subjects are summarized in Tables 1 and 2. All individuals undergoing liver transplantation were in advanced stages of liver disease.

**Fig 1:**
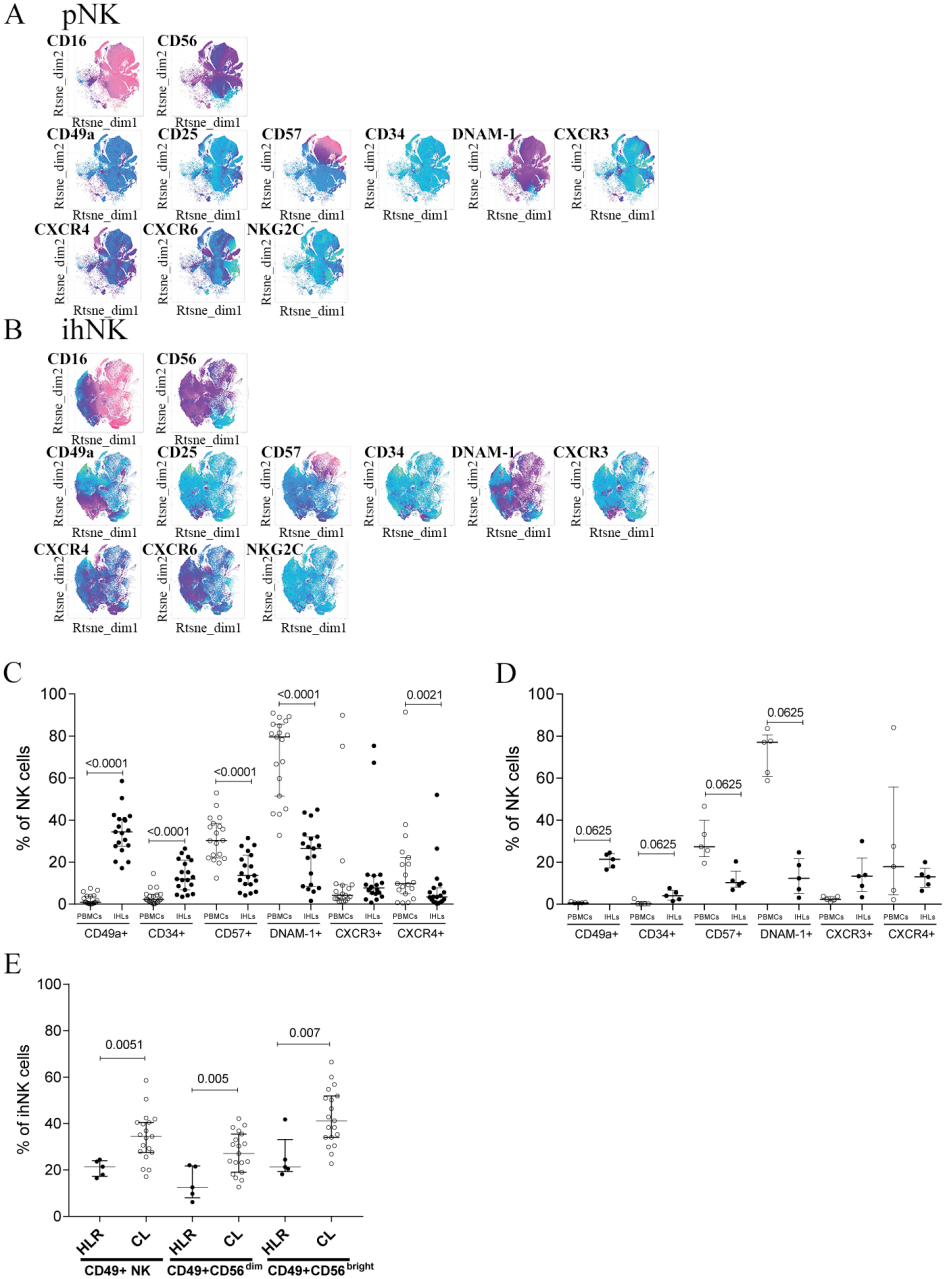
Immune phenotyping of combined peripheral and intrahepatic NK cells. Gated NK cells from 19 donors were concatenated and represented in t-SNE maps for the expression of chemokine receptors, activation and residency markers. **(A)** peripheral and **(B)** intrahepatic NK cells are shown. Color coding indicates the expression intensity of the surface marker, pink being higher expressed and green being lower expressed. **(C)** Proportion of NK cells derived from the liver (ihNK) and the peripheral blood (pNK) on the liver transplantation cohort expressing CD49a (pNK median (IQR): 0.9 (0.3–3.9); ihNK median (IQR): 34.4 (27.6–40.5); p<0.0001), CD34 (pNK median (IQR): 2.2 (1–4.7); ihNK median (IQR): 12 (6.8–20.9); p<0.0001), CXCR4 (pNK median (IQR): 9.8 (4.9–22.2); ihNK median (IQR): 3.4 (1.3–7.7); p = 0.0024), CD57 (pNK median (IQR): 19 (22–38.5); ihNK median (IQR): 13.7 (9.4–23.3); p<0.0001) and DNAM-1 (pNK median (IQR): 79.6 (51.5–85.6); ihNK median (IQR): 26.5 (8.5–32.1); p<0.0001) (n = 19). **(D)** Proportion of NK cells from the tumor-free liver resections expressing CD49a, CD34, CD57, DNAM-1, CXCR3 and CXCR4 within the IHLs NK cells and pNK cells (n = 5). **(E)** Frequency of CD49a+ NK cell population within the IHLs NK cells in tumor-free liver resection cohort (HLR) and the liver retransplant cohort (cirrhotic livers, CL). Data is depicted as scatter plot, with each dot corresponding to a participant. Bars indicate median and IQR. Wilcoxon signed rank tests with adjustment of p-values by false discovery rate.

The NK cell surface receptor repertoire between matched liver and peripheral blood samples was compared using the t-SNE dimensionality reduction algorithm, which provides two dimensional visualization of multiparametric single cell data [17]. To facilitate the comprehension of the final results, all 19 individuals undergoing liver transplantation were included in the same analysis. The results showed that the overall structure of peripheral blood NK (pNK) cells (Fig 1A) and intrahepatic NK (ihNK) cell (Fig 1B) repertoires differed since few plot regions were co-localizing in both groups. As expected, even the principal markers identifying NK cells, CD56 and CD16, exhibited differences. Confirming previous results, ihNK cells contained a higher proportion of cells expressing CD56 (CD56^bright^ NK cells) compared to pNK cells, [12]. Furthermore, CD49a expression was almost exclusively concentrated in ihNK cells and non-existent in pNK cells, demonstrating that CD49a enables differentiation of NK cells from these two compartments (Fig 1B). When focusing on the CD49a highly dense area, ihNK cells co-expressed cytokine receptors such as CXCR4, CXCR3 and CXCR6 as well as CD25 and CD34. Peripheral NK cells in contrast contained exclusively a defined area with high density of cells expressing maturation markers such as CD57 and DNAM-1 (Fig 1A).

We next sought to quantitatively confirm the visual t-SNE analysis results by statistical approaches. Matched intrahepatic and peripheral blood NK cell populations were manually gated and the frequency of each marker was individually quantified and compared (Fig 1C and 1D). Gating strategies are shown in S1 Fig. After adjustment for test multiplicity, CD56^bright^ NK cells were significantly more frequent and CD56^dim^ NK cells were significantly less frequent in the intrahepatic compartment compared to the peripheral blood compartment in the liver transplant cohort (S2A Fig), confirming previous studies [12]. Within the CD56-CD16+ cells, a previously described dysfunctional NK cell subset [25–27], no differences were detected between blood and liver NK cell frequencies. Furthermore, no phenotypical differences on NK cells were observed between liver diseases such as hepatitis C virus (HCV)-liver injury, autoimmune diseases (Primary sclerosing cholangitis (PSC) and Primary Biliary Cholangitis (PBC)), alcoholic liver disease (ALD), hepatocellular carcinoma (HCC) and polycystic liver disease in the studied cohort (data not shown), potentially due to small number of cases per disease (Table 1). The same tendency for NK cell subsets distribution was observed in the tumor-free areas in liver tumor resections (p = 0.0625) (S2B Fig), confirming previous studies [8] and the results from the t-SNE analysis. Compared to pNK cells, ihNK cells included a significantly higher proportion of NK cells expressing CD34, and a significantly lower proportion of NK cells expressing CD57 and DNAM-1, consistent with a less differentiated phenotype (Fig 1C). In line with previous data [6, 8], we confirmed results from the t-SNE plots showing that ihNK cells contained significantly higher frequencies of CD49a+ NK cells, a molecule that is not expressed in the peripheral blood NK cell compartment. In contrast, cells positive for the chemokine receptor CXCR4 were significantly less represented among the ihNK cells. The proportion of cells expressing the same markers in liver resections showed the same tendency (Fig 1D). The liver transplant cohort, which only contained cirrhotic livers, had a statistically higher frequency of CD49a+ ihNK cells when compared to tumor-free liver resections (p = 0.005) (Fig 1E). Overall, these data demonstrate that ihNK cells differ significantly from pNK cells, and exhibit a less differentiated phenotype. Moreover, cirrhosis and inflammation were associated with higher expression levels of CD49a on ihNK cells.

Similar data were observed when comparing peripheral blood and liver CD56^bright^ and CD56^dim^ NK cells (S2C and S2D Fig). The proportion of CD49a+ cells was significantly higher within the intrahepatic CD56^bright^ and CD56^dim^ NK cell populations than in matched peripheral blood NK cells. In contrast, CXCR4+ cells were significantly more frequent within peripheral CD56^dim^ and CD56^bright^ NK cells compared to ihNK cells. Taken together, these results suggest the presence of a more immature NK cell population in inflamed livers compared to peripheral blood.

### The liver-resident CD49a+ NK cell subset contains immature and pre-activated cells

**Fig 2:**
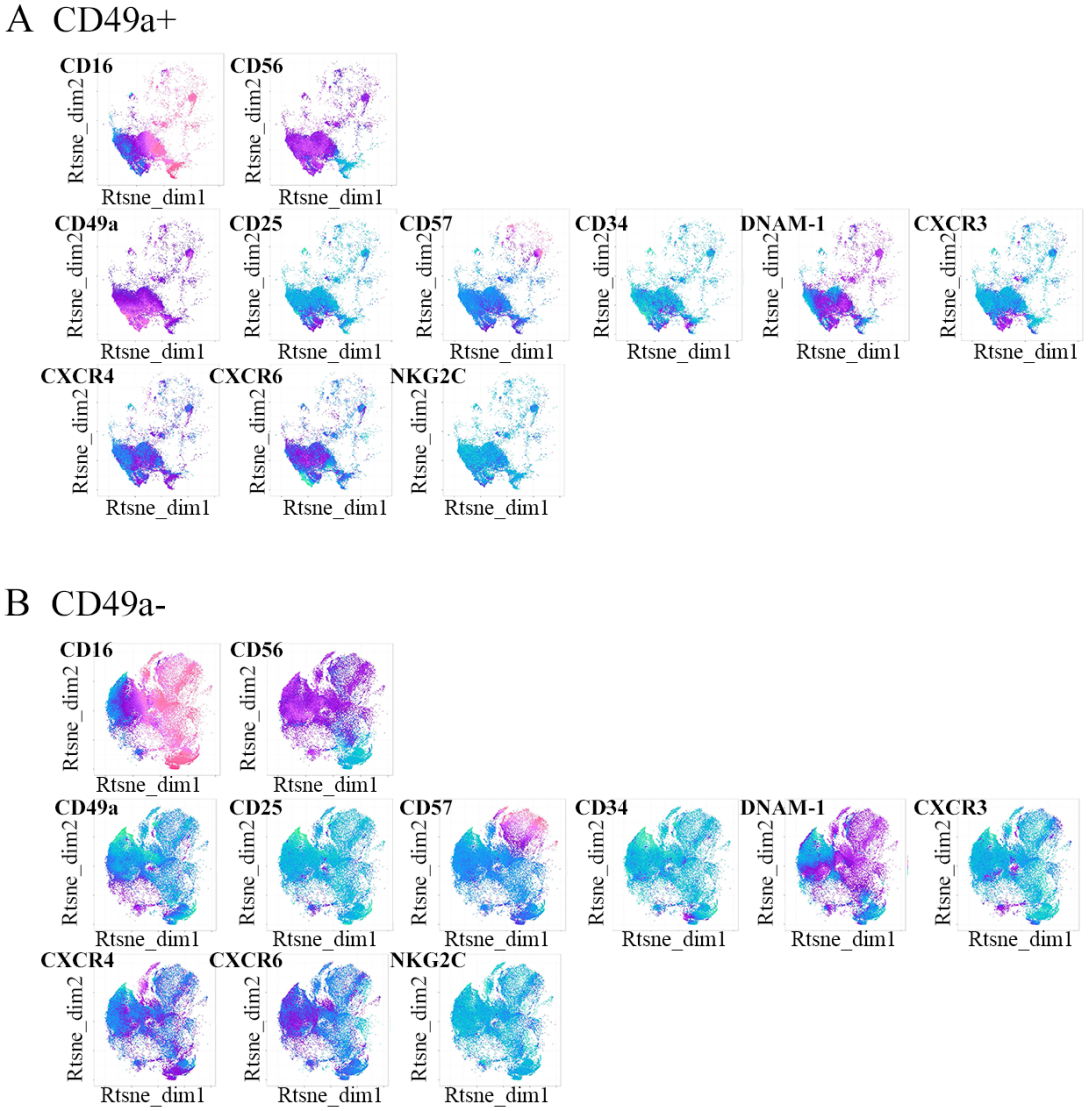
Unsupervised analysis of intrahepatic CD49a+ and CD49a- NK cells. **(A)** Gated CD49a+ and **(B)** CD49a- NK cells from 19 donors were concatenated and represented in t-SNE maps for the expression of chemokine receptors, activation and residency markers. Color coding indicates the expression intensity of the surface marker, pink being higher expressed and green being lower expressed.

CD49a (ITGA1, VLA-1) is an alpha 1 integrin binding to laminin and collagen, and has been shown to be expressed on liver-resident NK cells in mice and humans [6, 8]. We therefore investigated the phenotypical differences between intrahepatic CD49a^+^ and CD49a^-^ NK cells to further characterize this subset of liver-resident CD49a+ NK cells. CD49a+ and CD49a- NK cells showed distinct distributions within the t-SNE plots, already suggesting that these two ihNK cell populations possessed distinct phenotypical signatures (Fig 2). In particular, CXCR3, CXCR4, CD25 and CD34 were highly expressed in CD49a+ ihNK cells (Fig 2A), whereas CD49a- ihNK cells areas more closely resembled pNK cells (Figs 1A and 2B).

**Fig 3:**
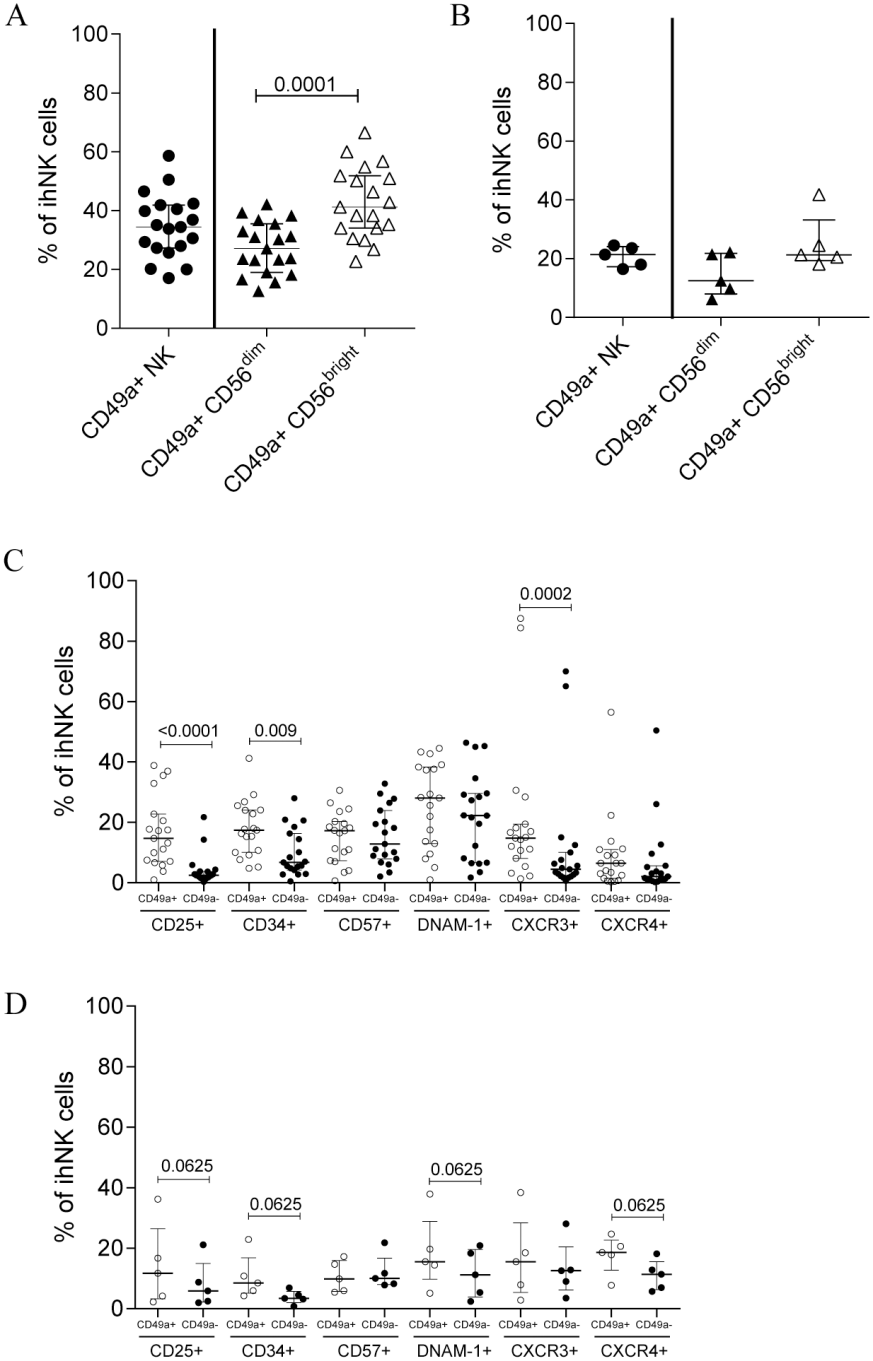
Immunophenotyping of intrahepatic CD49a+ and CD49a- NK cells. CD49a expression on bulk, CD56^dim^ and CD56^bright^ NK cells in the liver transplantation cohort **(A)** and in the tumor-free liver resection cohort **(B)**. Proportion of cells expressing specific markers in ihNK cells once gated on CD49a+ and CD49a- NK cells in the liver transplantation cohort **(C)** with CD25+ (CD49a+ NK cell median (IQR): 14.7 (7.1–22.7); CD49a- NK cell median (IQR): 2.5 (1.6–3.8); p<0.0001), CD34+ (CD49a+NK cell median (IQR): 17.4 (10–24.1); CD49a- NK cell median (IQR): 6.8 (4.2–16.3); p = 0.0107) and CXCR3+ (CD49a+ NK cell median (IQR): 14.8 (8.1–19.4); CD49a- NK cell median (IQR): 4.5 (2.2–10); p = 0.0002). **(D)** Proportion of cells expressing specific markers in ihNK cells once gated on CD49a+ and CD49a- in tumor-free liver resections. All data is depicted as scatter plot, with each dot corresponding to a participant. Bars indicate median and IQR. Wilcoxon signed rank tests with adjustment of p-values by false discovery rate.

When applying manual gating and statistical testing to differentiate between CD49a+ and CD49a- NK cells in livers, we observed that, although present in CD56^dim^ NK cells, CD49a+ cells were significantly more frequent within the CD56^bright^ NK cell population in the liver transplant cohort (p = 0.0001) (Fig 3A). In the tumor-free areas of liver resections, a similar trend was observed, but did not reach statistical significance (p = 0.185) (Fig 3B). The respective gating strategies are shown in S3 Fig. In the liver transplant cohort, intrahepatic CD49a+ NK cells furthermore differed from CD49a- NK cells by containing significantly higher proportions of CD25+, CD34+, and CXCR3+ cells (Fig 3C). No significant differences regarding the frequency of cells positive for maturation and activation markers CD57 and DNAM-1 as well as in CXCR4 expression were observed between CD49a+ and CD49a- intrahepatic NK cells. A similar trend was observed in the tumor-free areas of liver tumor resections (Fig 3D). These significant differences in the proportion of CD34, CD25 and CXCR3 between CD49a+ and CD49a- NK cells from the liver transplant cohort were observed both within the CD56^bright^ and CD56^dim^ NK cell subsets (S4 Fig).

### Liver-resident CD49a+CD25+ NK cells proliferate in response to low doses of IL-2

**Fig 4:**
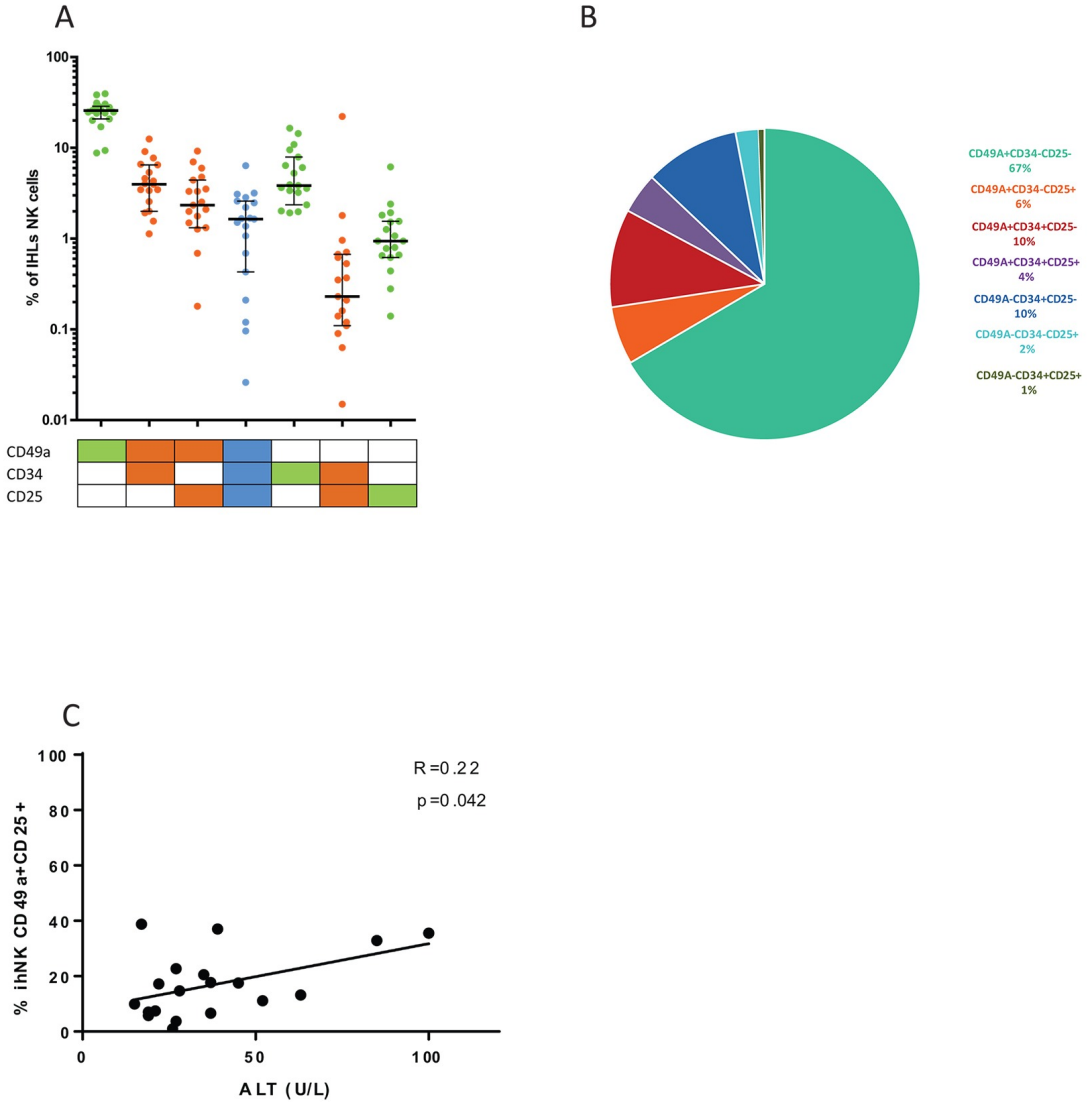
Relevance of CD49a+CD25+ ihNK cells. **(A)** Boolean gating of CD49a, CD25 and CD34 markers on ihNK cells. **(B)** Pie chart representing the frequency of each of the 7 possible combinations of the 3 markers, CD49a, CD25 and CD34. The median percentage of each population is represented. **(C)** Alanine Aminotransferase (ALT) serum levels correlation with the proportion of intrahepatic CD49a+CD25+ NK cells in the liver transplantation cohort. Data in **(A)** is depicted as scatter plot showing all individuals, the bar represents the median and the deviation is depicted as interquartile range.

The expression of CD34, a marker associated with hematopoietic stem cells [28, 29] by liver-resident CD49a+ NK cells, indicates potential for self-renewal and an immature phenotype. The high levels of CD25 expression, the high affinity receptor for IL-2, on liver-resident CD49a+ NK cells suggests proliferative capacities of these cells in response to cytokines in the context of liver inflammation. We therefore characterized the combined expression patterns of these markers in the context of CD49a on ihNK cells using Boolean gating, which revealed 7 possible distinct NK cell populations based on these three markers after excluding the triple negative (CD25^-^CD49a^-^CD34^-^) population of NK cells (Fig 4A). A pie chart representing the frequencies of each population normalized to 100% is shown to better visualize the contribution of each subset with regards to the three markers (Fig 4B). Boolean gating of the three markers confirmed that CD49a, expressed on 87% of liver-derived NK cells (excluding the triple negative population), was the main driver to differentiate ihNK cells (Fig 4B). Moreover, CD25 or CD34 were frequently co-expressed on CD49a+ NK cells (10.2% for CD49a+CD25-CD34+ cells, 6.03% for CD49a+CD25+CD34- cells), whereas single expression of CD25 or CD34 or combined expression of CD25 and CD34 in the absence of CD49a expression were less represented (9.9% for CD49a-CD34+CD25- cells, 2.4% for CD49a-CD34-CD25+ cells and 0.59% in CD49a-CD34+CD25+ cells) (Fig 4A and 4B).

To understand whether the presence of these subsets were related to high inflammation in the livers, Alanine Aminotransferase (ALT) serum levels were correlated to our flow cytometry data. ALT levels on the liver transplantation cohort positively correlated with the proportion of intrahepatic CD49a+CD25+ NK cells (Fig 4C).

**Fig 5:**
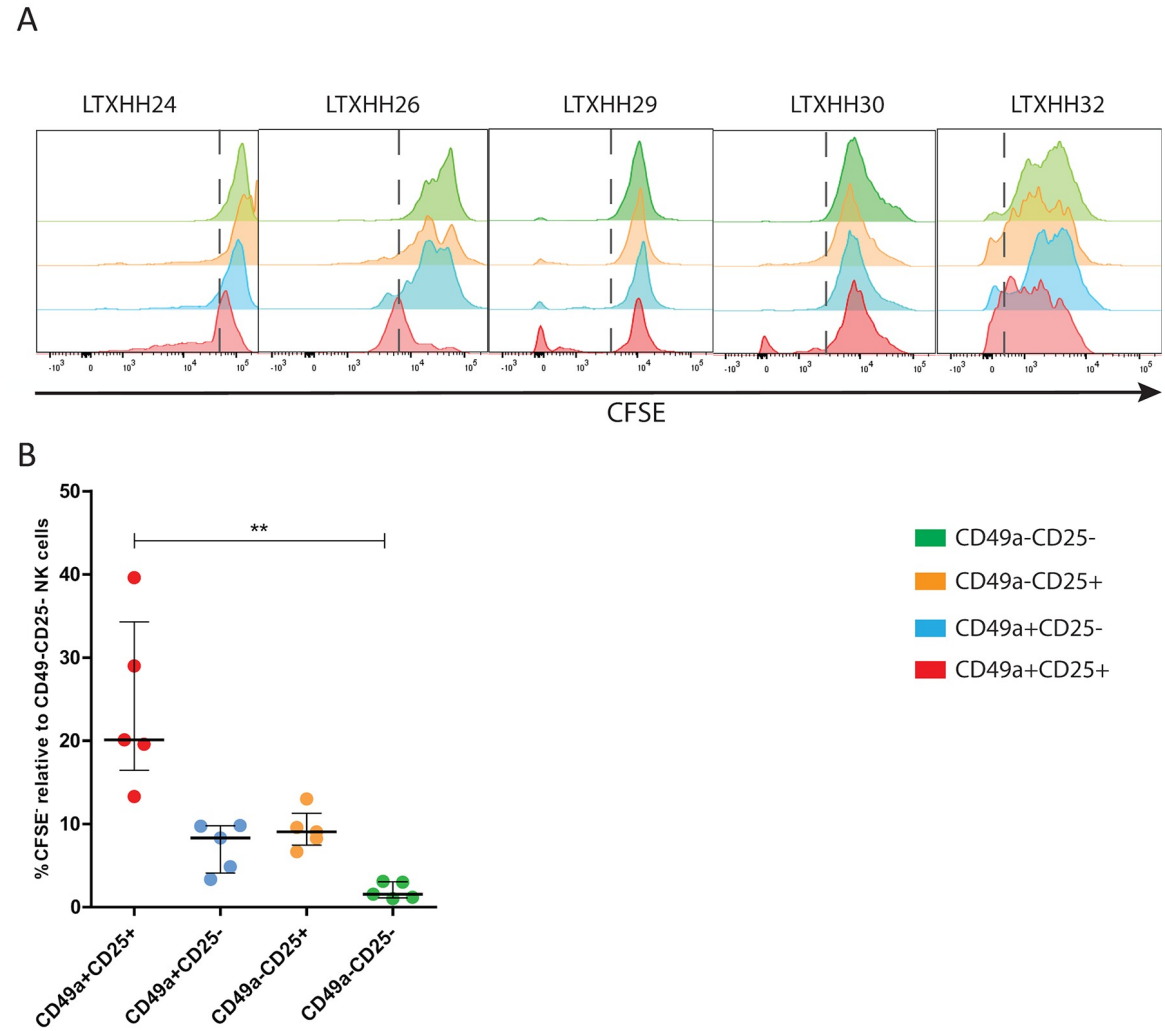
Functional assessment of liver-resident CD49a+ CD25 ihNK cells. **(A)** Representative histograms 7 days post-cell tracing with CFSE on 5 livers, LTXHH24, LTXHH26, LTXHH29, LTXHH30 and LTXHH32 (gated on live cells). Intrahepatic NK cells were sorted using a BD FACSARIA FUSION according to their expression levels of CD49a and CD25. Recovered cells were CFSE-cell stained and cultured for 7 days with low amounts of IL-2 (red: CD49a+CD25+, blue: CD49a+CD25-, orange: CD49a-CD25+, green: CD49a-CD25-). **(B)** Summary of the cell proliferation data from the four sorted populations. Percentage of CFSE+ cells was determined by setting the gate on the lower part of the CD49a-CD25- population (in green) as shown by the dashed lines. Data in **(B)** is depicted as scatter plot showing all individuals, the bar represents the median and the deviation is depicted as interquartile range.

CD25 is the high affinity receptor for IL-2, a cytokine overexpressed in inflammatory liver diseases [30], and showed a clear and almost exclusive expression on liver-resident CD49a+ NK cells. Since Boolean gating confirmed that CD34 and CD25 were almost mutually exclusively expressed markers, and CD49a+CD25+ ihNK cells were correlated to ALT levels, we focused on studying the functional properties of CD49a+CD25+ NK cells. CD49a+CD25+, CD49a+CD25-, CD49a-CD25+ and CD49a-CD25- NK cells were sorted from 5 livers, cell-traced with CFSE and cultured with low doses of IL-2 for 7 days (Fig 5A). Only CD49a+CD25+ cells were able to significantly proliferate in response to IL-2 when compared to other subsets (unadjusted p = 0.009) (Fig 5B). Taken together, these results show that CD49a+CD25+ NK cells are more prevalent in inflamed livers compared to blood, and have the ability to proliferate in response to low microenvironment doses of IL-2, potentially explaining their high prevalence in inflamed liver tissues.

## Discussion

Building evidence from mouse models and human samples has shown that liver-resident NK cells represent a specific cell population which can be identified by the expression of surface markers and transcription factors [6–12, 14, 15, 31, 32]. Specifically, it was demonstrated in mice that liver-restricted CD49a+DX5- NK cells, which also expressed high levels of CXCR6 and CD69, exhibit lower levels of CD62L and KLRG1 expression, indicating lower levels of maturation and proliferation [6, 33, 34]. Here, we compared human hepatic and peripheral NK cells and identified unique NK cell subsets in human livers which differentially expressed CD49a, CD25 and CD34 and differed in their proliferative capacity.

CD49a, an alpha-1 integrin binding to collagen and laminin, has been described in humans as a tissue-residency marker in CD56^bright^ NK cells derived from liver, uterus, decidua and tonsils [6, 8, 35–37]. In line with these studies, we observed that the CD56^bright^ NK cell compartment included a high proportion of CD49a+ cells in cirrhotic explant livers. While CD56^dim^ NK cells in these livers also contained CD49a+ cells, this was to a much lesser extent. In cirrhotic livers, the proportions of CD49+ cells in liver-derived CD56^bright^ and CD56^dim^ NK cells, were higher compared to the tumor-free liver resections. This high frequency of CD49a+ NK cells in liver-derived NK cells observed in the cirrhotic livers compared to liver resections might be in part a consequence of the advanced fibrosis stages in the liver transplant samples investigated. Early studies have shown that advanced stages of liver fibrosis are associated with the accumulation of hepatic extracellular matrix (ECM) secreted by hepatic stellate cells (HSCs) [38]. ECM is rich in fibrillary collagens, which act as a ligand for CD49a [39–42] and CD49a+ NK cells might therefore be preferentially recruited to inflamed and fibrotic livers and accumulate in these tissues.

The factors that enable CD49a+ NK cells to persist or expand in livers remain unknown. Recently, it was described that CD56^bright^CD25+ decidual NK cells are trafficked towards the maternal/fetal interface in early stages of pregnancy depending on CXCR4 expression [43]. In line with those results, we observed that liver-resident CD49a+ NK cells harbored high proportions of cells expressing CD25. CD25 was not present on peripheral blood NK cells in our study, which is in line with previous studies [44]. CD49a+CD25+ NK cells exhibited a higher proliferative capacity *in vitro* compared to CD49a-CD25+, CD49a-CD25- and CD49a+CD25- NK cells when stimulated with low doses of IL-2. These data suggested that liver-resident NK cells expressing CD25 have a lower activation threshold for IL-2 stimulation when compared to non-liver-resident NK cells, potentially enabling these cells to locally expand during inflammation and cirrhotic processes. A recent study demonstrated in liver biopsies that IL-2 protein levels were significantly higher (almost 5 times) in ALD and PBC samples compared to HCV-induced cirrhotic livers and healthy liver tissue [30]. Nonetheless, and potentially due to the small amount of samples per disease, the presence of CD49a+CD25+ ihNK cells in the liver transplantation cohort was not significantly different when comparing different diseases settings. During inflammatory processes, IL-2 is synthesized in secondary lymphoid organs, such as lymph nodes, primarily by CD4+ T helper (T_H_) cells, but also CD8+ T cells, NK cells and dendritic cells (DCs) contribute to its production [44–48]. In liver diseases, the production of IL-2 is increased in liver tissues as well as in serum (soluble IL-2), and is considered a biomarker for poor prognosis [49, 50]. In response to liver injury, Kupffer cells (KCs) produce IL-15, IL-12, IL-18 and TNFα to modulate the survival and activity of NK cells [51]. Additionally, overexpression of CD25 on NK cells has been described *in vitro* to be triggered by IL-15 plus IL-18 or IL-12 plus IL-18, increasing their functionality and proliferative capacity in response to picomolar concentrations of IL-2 [52]. In summary, our data suggests that CD25 expression by liver-resident CD49a+ NK cells might be a response to the inflammatory cytokines produced in inflamed livers.

Early studies described the presence of Pluripotent Stem Cells (PSCs) in liver adult mice models [53]. During human NK cell development, CD34 is expressed on NK cell precursors up to stage 2, a versatile precursor of T, DC and NK cells. In stage 3, NK cells loose CD34 expression and are already defined as committed NK cell development intermediates, as they are not able to mature into other immune cells, such as T or DC cells, *in vitro* [29, 54]. Hematopoiesis has shown to take place in the liver during the first trimester of fetal development [55, 56]. Using healthy human livers, previous studies demonstrated the presence of hematopoietic precursor cells bearing CD34 and CD45, with approximatively 37% of these CD34+ cells also expressing CD56 [56, 57]. Human hepatic NK cell progenitors were suggested to be recruited from the circulating peripheral lymphocyte population and were capable to differentiate into functional mature NK cells [58]. In line with these studies, we observed the presence of CD34+ NK cells exclusively within the CD49a+ ihNK cell population, representing around 18% of the total CD49a+ NK cell population. Using Boolean gating, we showed that the simultaneous presence of CD34 and CD25 on CD49a+ NK cells was low (1.64%), indicating that a very small population of NK cell progenitors would immediately respond to low concentrations of IL-2. All together, our results suggested two plausible hypotheses to explain the presence of CD49a+CD34+CD25- NK cells. Peripheral CD34+ NK cells might be recruited in the adult inflamed liver, and expression of CD49a in response to liver collagen retains them in the tissue to generate novel populations of NK cells. Alternatively, CD49a+CD34+CD25- NK cells might be retained in the liver tissue since fetal hematopoiesis, and might play a role in the generation of NK cell progenitors in adult liver. Whether CD49a+CD34+CD25- NK cells originate in the liver or are recruited to it remains to be elucidated.

In the liver, CXCR3-ligands are produced by hepatic sinusoidal endothelial cells, by activated KCs and by infiltrating leukocytes in response to IFNγ and TNFα stimulation [59–63]. In chronic liver diseases, the interferon gamma inducible protein-10 (IP-10), MIG and I-TAC, ligands for CXCR3, are overexpressed in liver tissues, leading to the recruitment of effector cells towards the liver. It has been suggested that liver-homing NK cells express high levels of CXCR3 in response to IP-10 microenvironment concentrations and that CD56^bright^CXCR3+ NK cells are functionally impaired and expanded in peripheral blood [64]. Our results using inflamed explant liver tissues demonstrate that CXCR3 was indeed significantly upregulated on CD49a+ liver-resident NK cells.

In conclusion, our data show that intrahepatic NK cells differ phenotypically from peripheral NK cells in individual-matched samples and that liver-resident CD49a+ NK cells additionally express exclusively CD25 or CD34. Indeed, the presence of liver-resident CD49a+CD34+ and CD49a+CD25+ NK cells demonstrate different phenotypical and functional features of liver-resident NK cells compared to peripheral blood NK cells including potential self-renewal and persistence.

## Supporting information

S1 Fig Gating strategy for the identification of ihNK (A) and pNK (B) cells. Representative contour plot for the identification of NK cells from liver samples. Lymphocytes were identified with CD45 after an initial gating on Forward (FCS-Area) and Sideward Scatter (SSC-Area) with a subsequent exclusion of doublets (FSC Width and SSC Width). NK cells were defined as CD3-CD14-CD19-CD56+CD16+/- lymphocytes. Zombie aqua was used for the exclusion of dead cells.
(TIF)

S2 Fig Immunophenotyping of liver and peripheral NK cells. Proportion of CD56^dim^, CD56^bright^ and CD16^+^CD56^-^ NK cells within the intrahepatic and peripheral blood NK cells compartment in **(A)** liver transplantation cohort and **(B)** tumor-free resection cohort. (**C, D**) CD56^bright^ and CD56^dim^ NK cells immunophenotyping from the liver transplantation cohort with the shown markers. **(C)** On CD56^bright^ NK cells, the following markers were observed: CD49a (CD56^bright^ pNK median (IQR): 3.2 (1.5–6.9); CD56^bright^ ihNK median (IQR): 41.2 (34.1–50.9); p < 0.0001), CD34 (CD56^bright^ pNK median (IQR): 4.1 (1.3–6.2); CD56^bright^ ihNK median (IQR): 10.8 (4.5–16.8); p = 0.0008), DNAM-1 (CD56^bright^ pNK median (IQR): 74.8 (56.7–87.5); CD56^bright^ ihNK median (IQR): 15.6 (7.6–20.8); p<0.0001) and CXCR4 (CD56^bright^ pNK median (IQR): 17 (1.4–26.6); CD56^bright^ ihNK median (IQR): 3.2 (0.6–6.7); p = 0.0004) when comparing CD56^bright^ ihNK and pNK cells. **(D)** Similarly, on CD56^dim^ NK cells, the following markers were observed: CD49a (CD56^dim^ pNK median (IQR): 0.4 (0.2–2.8); CD56^dim^ ihNK median (IQR): 27.1 (19–35.3); p < 0.0001), CD34+ cells (CD56^dim^ pNK median (IQR): 1.9 (0.4–4.2); CD56^dim^ ihNK median (IQR): 27.1 (5.1–14.2); p = 0.0001), DNAM-1+ cells (CD56^dim^ pNK median (IQR): 82.2 (51.9–77.4); CD56^dim^ ihNK median (IQR): 51.1 (26.1–67); p = 0.0001) and CXCR4+ cells (CD56^dim^ pNK median (IQR): 7.8 (2.9–22); CD56^dim^ ihNK median (IQR): 2.8 (1.5–5.6); p = 0.0024) when comparing CD56^dim^ ihNK and pNK cells. Data is depicted as scatter plot, with each dot corresponding to a participant. Bars indicate median and IQR. Wilcoxon signed rank tests with adjustment of p-values by false discovery rate.
(TIF)

S3 Fig Gating strategy of intrahepatic (A) CD49a+ and (B) CD49a- NK cells for CD25, CXCR3 and CD34 markers. Following the identification shown in S1 Fig, characterization of **(A)** CD49a+ and **(B)** CD49a- was performed. Representative contour plots are shown.
(TIF)

S4 Fig Immunophenotyping of intrahepatic CD49a+ and CD49a+- NK cells derived from the liver transplantation cohort on CD56^bright^ and CD56^dim^ NK cells. **(A)** CD56^bright^ ihNK showed the following proportions for CD25+ (CD49a+CD56^bright^ NK cell median (IQR): 13.5 (7.3–26.3); CD49a- CD56^bright^ NK cell median (IQR): 2.3 (1.9–7.7); p<0.0001), CD34+ (CD49a+ CD56^bright^ NK cell median (IQR): 15.4 (8.5–22.7); CD49a-CD56^bright^ NK cell median (IQR): 4.7 (3.4–14.2); p = 0.0030) and CXCR3+ (CD49a+ CD56^bright^ NK cell median (IQR): 15.6 (11.8–29.6); CD49a- CD56^bright^ NK cell median (IQR): 4.8 (3.1–14); p = 0.0004) in CD49a+ ihNK cells when compared to CD49a- ihNK cells. **(B)** As for CD56^dim^ NK cells, the data also displayed the following proportions of CD25+ (CD49a+CD56^dim^ NK cell median (IQR): 12.4 (7.5–23.4); CD49a- CD56^dim^ NK cell median (IQR): 2.4 (1.9–3.9); p<0.0001), CD34+ (CD49a+CD56^dim^ NK cell median (IQR): 14.8 (9.6–23.5); CD49a- CD56^dim^ NK cell median (IQR): 6 (4.2–14.7); p = 0.0027), and CXCR3+ (CD49a+CD56^dim^ NK cell median (IQR): 7 (2.2–15.1); CD49a- CD56^dim^ NK cell median (IQR): 2.4 (1.1–6.2); p = 0.0184) cells in the CD49a+ intrahepatic subset compared to the CD49a- intrahepatic subset. Data is depicted as scatter plot, with each dot corresponding to a participant. Bars indicate median and IQR. Wilcoxon signed rank tests with adjustment of p-values by false discovery rate.
(TIF)
