# Supplementary figures and images for: Proliferative capacity exhibited by human liver-resident CD49a+CD25+ NK cells
Source: PLoS One. 2017 Aug 9;12(8):e0182532. doi: 10.1371/journal.pone.0182532 (PMC5549915; doi:10.1371/journal.pone.0182532)

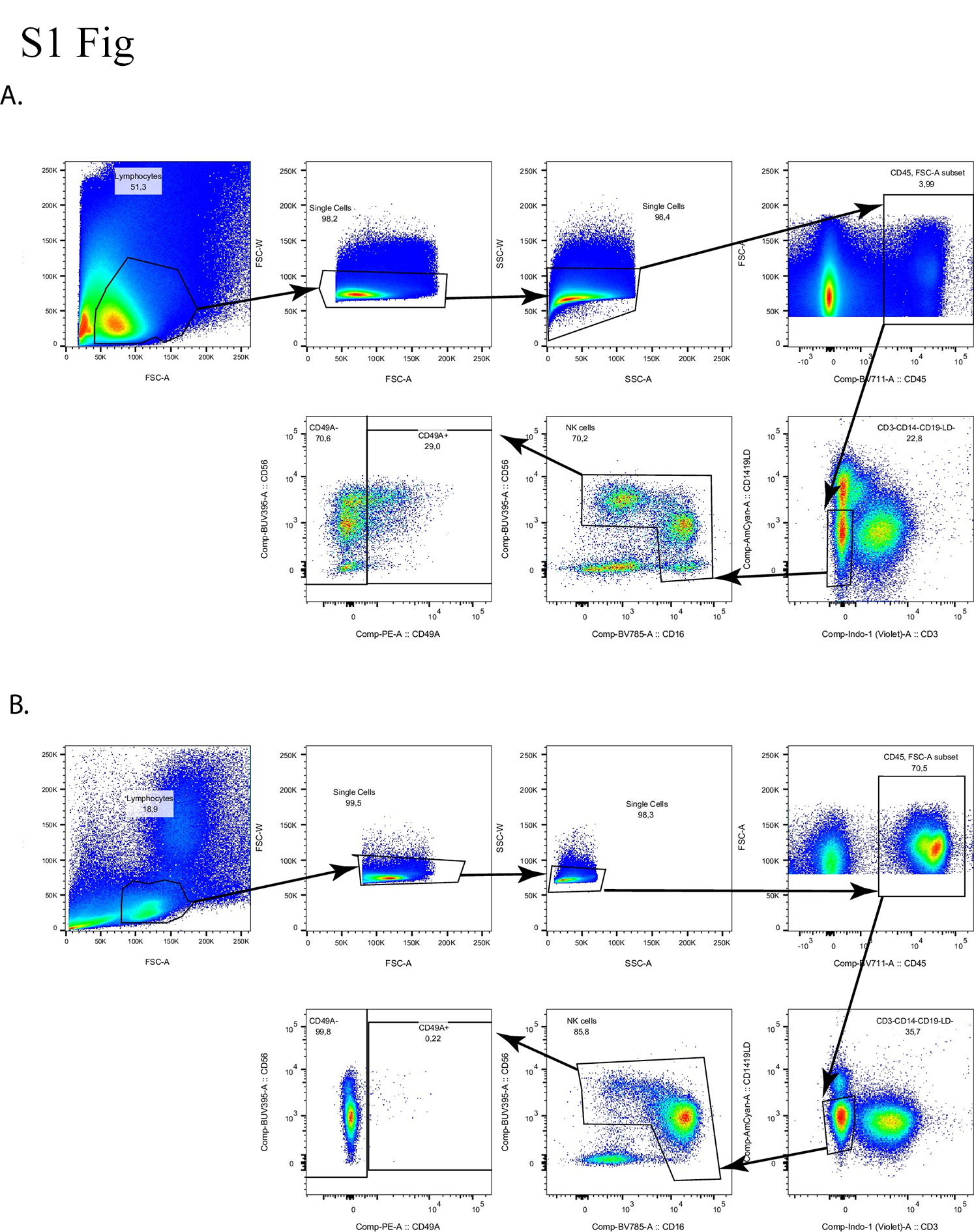

Supplement: S1 Fig — Representative contour plot for the identification of NK cells from liver samples. Lymphocytes were identified with CD45 after an initial gating on Forward (FCS-Area) and Sideward Scatter (SSC-Area) with a subsequent exclusion of doublets (FSC Width and SSC Width). NK cells were defined as CD3-CD14-CD19-CD56+CD16+/- lymphocytes. Zombie aqua was used for the exclusion of dead cells. (TIF) [file pone.0182532.s001.tif]

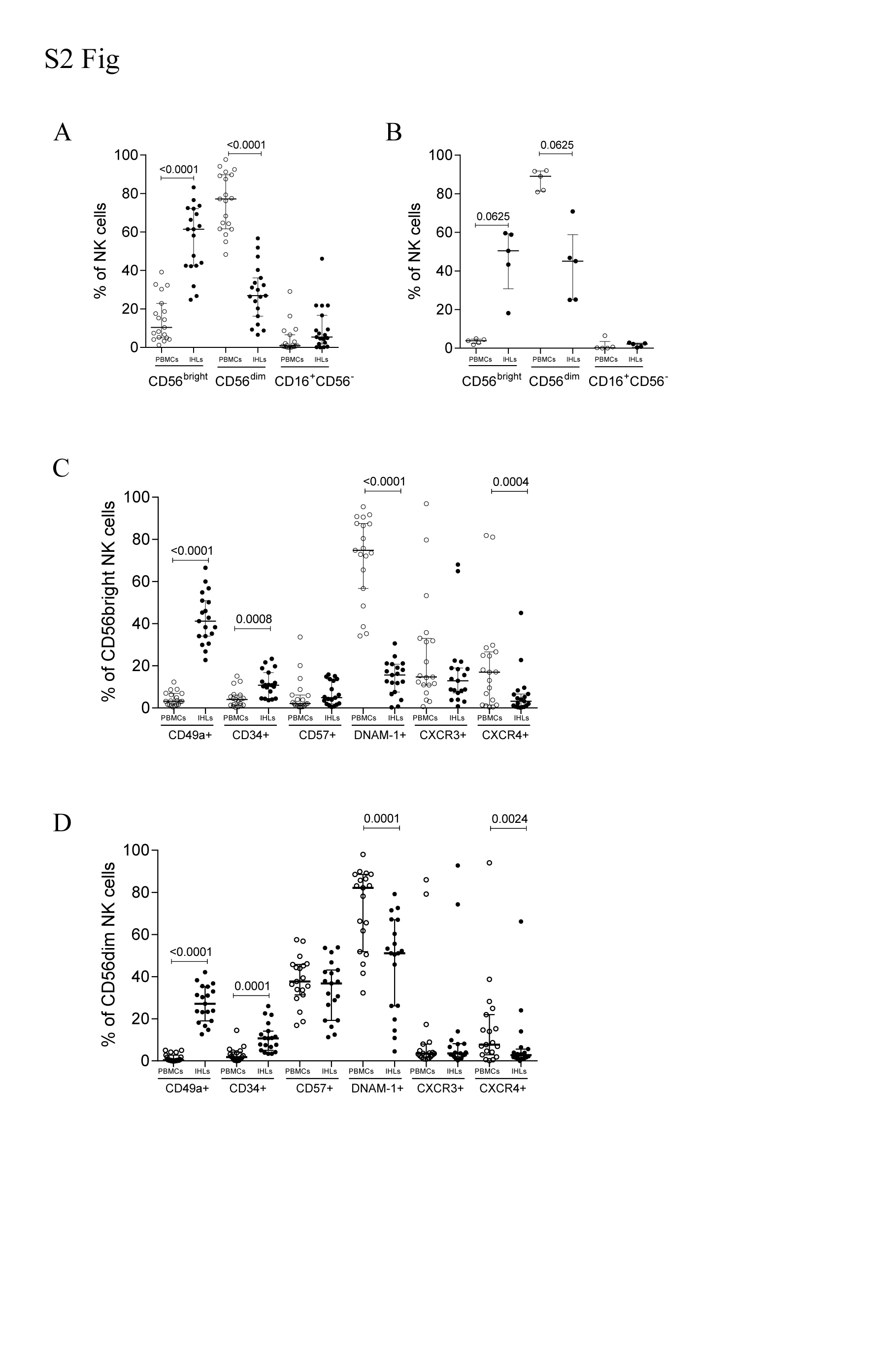

Supplement: S2 Fig — Proportion of CD56dim, CD56bright and CD16+CD56- NK cells within the intrahepatic and peripheral blood NK cells compartment in (A) liver transplantation cohort and (B) tumor-free resection cohort. (C, D) CD56bright and CD56dim NK cells immunophenotyping from the liver transplantation cohort with the shown markers. (C) On CD56bright NK cells, the following markers were observed: CD49a (CD56bright pNK median (IQR): 3.2 (1.5–6.9); CD56bright ihNK median (IQR): 41.2 (34.1–50.9); p < 0.0001), CD34 (CD56bright pNK median (IQR): 4.1 (1.3–6.2); CD56bright ihNK median (IQR): 10.8 (4.5–16.8); p = 0.0008), DNAM-1 (CD56bright pNK median (IQR): 74.8 (56.7–87.5); CD56bright ihNK median (IQR): 15.6 (7.6–20.8); p<0.0001) and CXCR4 (CD56bright pNK median (IQR): 17 (1.4–26.6); CD56bright ihNK median (IQR): 3.2 (0.6–6.7); p = 0.0004) when comparing CD56bright ihNK and pNK cells. (D) Similarly, on CD56dim NK cells, the following markers were observed: CD49a (CD56dim pNK median (IQR): 0.4 (0.2–2.8); CD56dim ihNK median (IQR): 27.1 (19–35.3); p < 0.0001), CD34+ cells (CD56dim pNK median (IQR): 1.9 (0.4–4.2); CD56dim ihNK median (IQR): 27.1 (5.1–14.2); p = 0.0001), DNAM-1+ cells (CD56dim pNK median (IQR): 82.2 (51.9–77.4); CD56dim ihNK median (IQR): 51.1 (26.1–67); p = 0.0001) and CXCR4+ cells (CD56dim pNK median (IQR): 7.8 (2.9–22); CD56dim ihNK median (IQR): 2.8 (1.5–5.6); p = 0.0024) when comparing CD56dim ihNK and pNK cells. Data is depicted as scatter plot, with each dot corresponding to a participant. Bars indicate median and IQR. Wilcoxon signed rank tests with adjustment of p-values by false discovery rate. (TIF) [file pone.0182532.s002.tif]

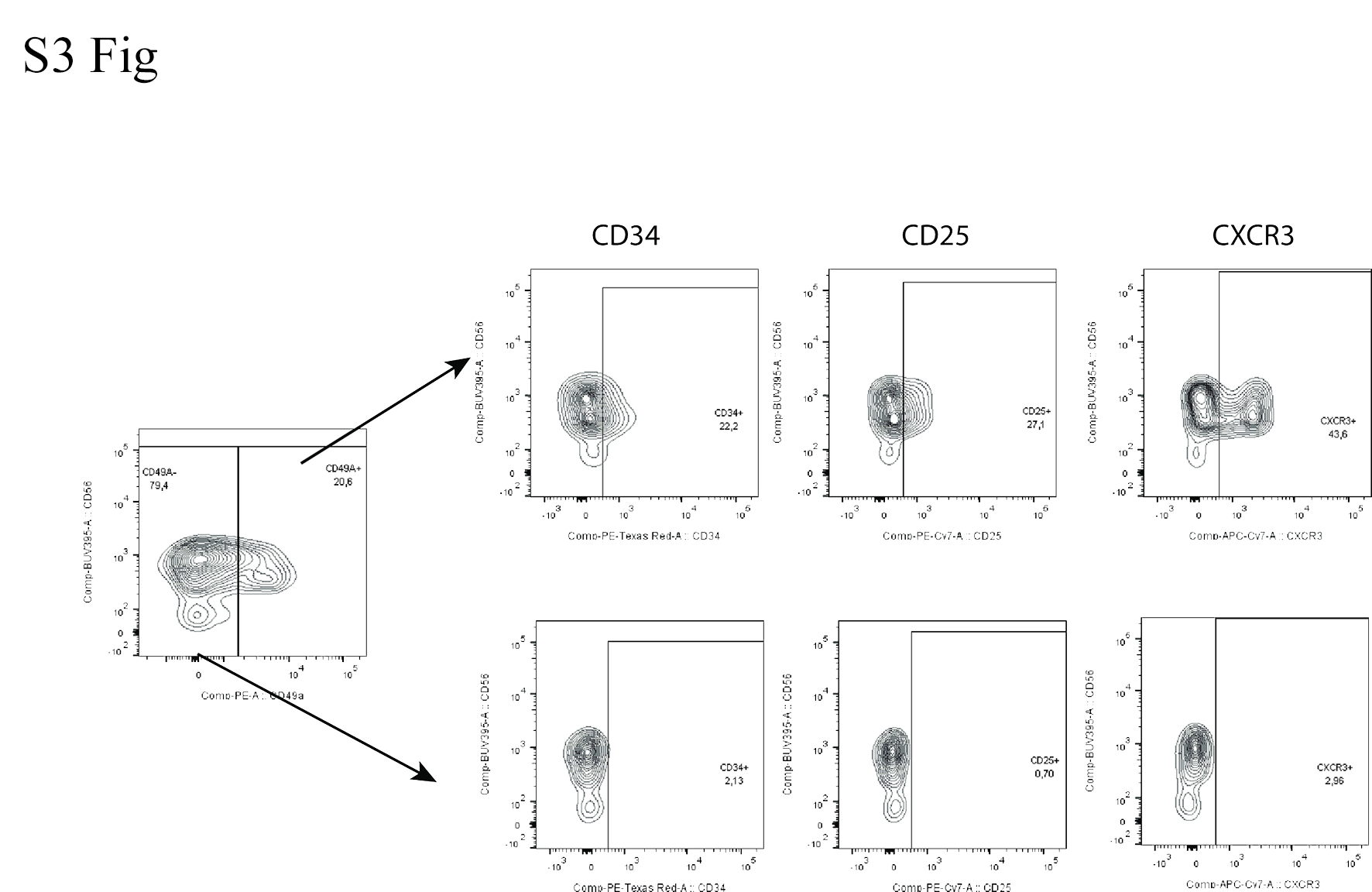

Supplement: S3 Fig — Following the identification shown in S1 Fig, characterization of (A) CD49a+ and (B) CD49a- was performed. Representative contour plots are shown. (TIF) [file pone.0182532.s003.tif]

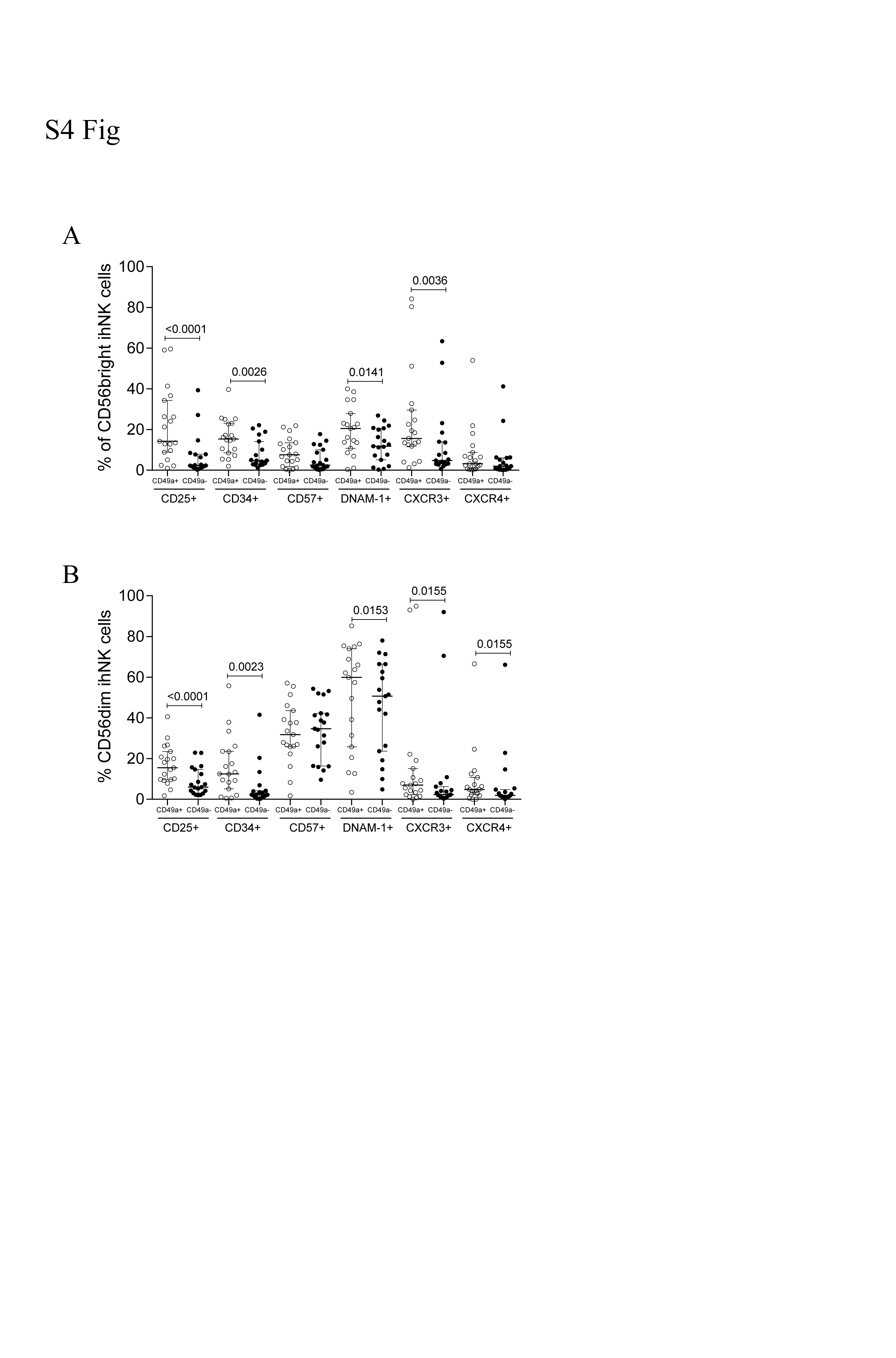

Supplement: S4 Fig — (A) CD56bright ihNK showed the following proportions for CD25+ (CD49a+CD56bright NK cell median (IQR): 13.5 (7.3–26.3); CD49a- CD56bright NK cell median (IQR): 2.3 (1.9–7.7); p<0.0001), CD34+ (CD49a+ CD56bright NK cell median (IQR): 15.4 (8.5–22.7); CD49a-CD56bright NK cell median (IQR): 4.7 (3.4–14.2); p = 0.0030) and CXCR3+ (CD49a+ CD56bright NK cell median (IQR): 15.6 (11.8–29.6); CD49a- CD56bright NK cell median (IQR): 4.8 (3.1–14); p = 0.0004) in CD49a+ ihNK cells when compared to CD49a- ihNK cells. (B) As for CD56dim NK cells, the data also displayed the following proportions of CD25+ (CD49a+CD56dim NK cell median (IQR): 12.4 (7.5–23.4); CD49a- CD56dim NK cell median (IQR): 2.4 (1.9–3.9); p<0.0001), CD34+ (CD49a+CD56dim NK cell median (IQR): 14.8 (9.6–23.5); CD49a- CD56dim NK cell median (IQR): 6 (4.2–14.7); p = 0.0027), and CXCR3+ (CD49a+CD56dim NK cell median (IQR): 7 (2.2–15.1); CD49a- CD56dim NK cell median (IQR): 2.4 (1.1–6.2); p = 0.0184) cells in the CD49a+ intrahepatic subset compared to the CD49a- intrahepatic subset. Data is depicted as scatter plot, with each dot corresponding to a participant. Bars indicate median and IQR. Wilcoxon signed rank tests with adjustment of p-values by false discovery rate. (TIF) [file pone.0182532.s004.tif]
